# RETRACTED: Kim et al. The p53-Driven Anticancer Effect of *Ribes fasciculatum* Extract on AGS Gastric Cancer Cells. *Life* 2022, *12*, 303

**DOI:** 10.3390/life16060963

**Published:** 2026-06-08

**Authors:** Myeong-Jin Kim, Hye-Won Kawk, Sang-Hyeon Kim, Hyo-Jae Lee, Ji-Won Seo, Chang-Yeol Lee, Young-Min Kim

**Affiliations:** Department of Biological Science and Biotechnology, College of Life Science and Nano Technology, Hannam University, Deajoen 34054, Republic of Korea; jiny2415@naver.com (M.-J.K.); gyp03416@daum.net (H.-W.K.); ssho26@naver.com (S.-H.K.); canopus310@naver.com (H.-J.L.); ddongji28@naver.com (J.-W.S.); jefferysun4@naver.com (C.-Y.L.)

The journal retracts the article titled, “The p53-Driven Anticancer Effect of *Ribes fasciculatum* Extract on AGS Gastric Cancer Cells” [1], cited above.

Following publication, concerns were brought to the attention of the Editorial Office regarding the integrity of a figure presented in this article [1].

In accordance with the journal’s standard procedures, an investigation was conducted by the Editorial Office and Editorial Board that identified indications of inappropriate editing in Figure 4A and duplication in Figure 6. Despite repeated attempts to contact the authors, no response was received. Consequently, the Editorial Board has lost confidence in the reliability of the reported findings and has decided to retract this publication, as per MDPI’s retraction policy.

This retraction was approved by the Editor-in-Chief of the journal *Life*.

The authors have been informed of this retraction but did not provide a comment on this decision.
